# Unlocking the Gateway: The Spatio-Temporal Dynamics of the p53 Family Driven by the Nuclear Pores and Its Implication for the Therapeutic Approach in Cancer

**DOI:** 10.3390/ijms25137465

**Published:** 2024-07-07

**Authors:** Dini Kurnia Ikliptikawati, Kei Makiyama, Masaharu Hazawa, Richard W. Wong

**Affiliations:** 1 Cell-Bionomics Research Unit, Innovative Integrated Bio-Research Core, Institute for Frontier Science Initiative, Kanazawa University, Kakuma-machi, Kanazawa 9201192, Japan; mhazawa@staff.kanazawa-u.ac.jp; 2 Laboratory of Molecular Cell Biology, Division of Transdisciplinary Sciences, Graduate School of Frontier Science Initiative, Kanazawa University, Kakuma-machi, Kanazawa 9201192, Japan; 3 WPI Nano Life Science Institute (WPI-NanoLSI), Kanazawa University, Kakuma-machi, Kanazawa 9201192, Japan

**Keywords:** p53 family, stabilization, degradation, nuclear pore complex, cancer, therapeutic

## Abstract

The p53 family remains a captivating focus of an extensive number of current studies. Accumulating evidence indicates that p53 abnormalities rank among the most prevalent in cancer. Given the numerous existing studies, which mostly focus on the mutations, expression profiles, and functional perturbations exhibited by members of the p53 family across diverse malignancies, this review will concentrate more on less explored facets regarding p53 activation and stabilization by the nuclear pore complex (NPC) in cancer, drawing on several studies. p53 integrates a broad spectrum of signals and is subject to diverse regulatory mechanisms to enact the necessary cellular response. It is widely acknowledged that each stage of p53 regulation, from synthesis to degradation, significantly influences its functionality in executing specific tasks. Over recent decades, a large body of data has established that mechanisms of regulation, closely linked with protein activation and stabilization, involve intricate interactions with various cellular components. These often transcend canonical regulatory pathways. This new knowledge has expanded from the regulation of genes themselves to epigenomics and proteomics, whereby interaction partners increase in number and complexity compared with earlier paradigms. Specifically, studies have recently shown the involvement of the NPC protein in such complex interactions, underscoring the further complexity of p53 regulation. Furthermore, we also discuss therapeutic strategies based on recent developments in this field in combination with established targeted therapies.

## 1. Overview of the p53 Family

The p53 family, including p53, p63, and p73, has basically been implicated in cellular life as a transcriptional powerhouse. Accumulating evidence accentuates the high importance of the functional nature of p53 inactivation in carcinogenesis and drug resistance. These high impacts emanate from the high complexity of the functions of the family of p53 that exhibit a great diversity. The p53 family controls not only multiple cellular fates at the single-cell level but also shapes cellular identity, determines the stage of the cell cycle, and activates or inhibits stress-induced survival or death programs. This is why developing a broader perspective in the understanding of p53 mechanisms is of prime significance. To achieve the complete picture, researchers are challenged to unravel not only how p53 family members with their various isoforms function as main factors in a canonical regulatory network but also how p53 can engage in non-canonical roles through genomic, epigenomic, and proteomic interactions [1,2,3,4].

Evolutionary conservation between p53 family members is reflected in their remarkable structural similarity across three of the most important and functionally relevant domains: the transactivation domain (TAD), the core/DNA-binding domain (DBD), and the oligomerization/tetramerization domain (OD/TD). An interesting feature, the SAM domain, based on five helices in total (four regular α-helices and one 310-helix) has been shown to be found in p63 and p73 but not p53 [5]. Intriguingly, the OD/TD is flanked by intrinsically disordered regions, facilitating cross-regulation of their target genes involved in key cellular metabolism including the cell cycle, apoptosis, autophagy, and DNA repair [4,6,7,8,9]. In the DBD of p53, more than 90% of mutations are point mutations, contributing to up to 50% of all cancer cases. These mutations result in a protein with diminished ability to bind to a particular DNA sequence, affecting the regulation of the p53 transcriptional pathway. Conversely, mutations in p63 and p73 are rare occurrences in cancer [10,11,12].

Additional complexities emerge as these p53 family proteins give rise to diverse isoforms through the use of distinct promoters, alternative splicing processes, and the internal ribosome entry site (IRES). These isoforms may act either in an antagonistic way or in a concerted way with the action of p53. The cross-talk between these different isoforms, mediated by the hetero-oligomerization formed by mixed tetramerization, is proposed to be a major determinant controlling the net activity of all the p53 family members. Expression of p53 isoforms was shown to be tissue- and context-specific for each isoform [6,13,14]. The p53 family genes encode essentially two types of isoforms, TA isoforms and ΔN isoforms, with or lacking TAD, respectively. The former act as tumor suppressors while the latter usually play oncogenic roles by blocking apoptosis and stimulating cell proliferation; meanwhile, ΔN isoforms can reduce TA isoform abundance and its transcriptional functions. The above results have already emphasized differential expression levels of the TA and ΔN isoforms from one cancer type to another, which are primarily responsible for governing tumor behavior, and thus, dictating clinical outcome, indicating that awareness of frequent functions of such isoforms is crucial for rational therapy and biomarker development [15,16,17,18].

The potent cytotoxic effects induced by p53 activity prompt cellular compensation to restrict its actions through the positive and negative regulatory (feedback loops) mechanisms in both physiological and pathological (cancerous) contexts [3,19]. As a negative autoregulator, a p53-responsive E3 ubiquitin ligase, MDM2, antagonizes p53 by inducing the ubiquitination and subsequent proteasomal degradation, "masking" its transactivation domain, promoting cytosolic accumulation, and inhibiting mRNA translation by disrupting the interaction between the ribosomal protein RPL26 with *TP53* mRNA [20]. RPL26 also plays a crucial role in the splicing of *TP53* pre-mRNA and the production of specific mRNA isoforms in human cancer cells [21,22]. MDM2 is frequently overexpressed in numerous cancers, causing non-mutational inactivation of p53 [23,24,25]. On the other hand, MDM2 insufficiency leads to p53-mediated apoptosis, as has been shown in in vivo studies [26]. These facts demonstrate the critical role of MDM2 in p53 cellular homeostasis. Although not as well studied as MDM2’s interaction with wild-type p53, studies have suggested both mutant forms of p53 along with p73 bind strongly to MDM2, whereas p63 demonstrated significantly lower affinity [3,27,28,29]. Cancers can arise either due to the adverse activities of the mutant p53s residing in the chromatin or by the impairment of the entire p53 signaling pathway. The integration of many other signals, genomic, epigenomic, and proteomic, to dictate conformation in p53 suggests that this protein exhibits various functionalities, aside from its canonical role as a transcriptional factor, making it complicated and versatile in its tumor-suppressive and homeostatic activities [30].

More than 60% of the sequence identity is shared between the DBD of p53 and its homologs, p63 and p73. Also, these are expressed in multiple isoforms, across various tissues and tumors [5,31]. p63 is essential for the development, differentiation, and homeostasis of human epithelial cells [31,32]. However, similar to p53, p73 not only inhibits cell growth but also coordinates many other cellular processes, including cytoskeletal dynamics [33,34]. The full-length versions, TAp63 and TAp73, are generally considered tumor suppressors. Mutant p53 proteins inhibit TAp63 and TAp73 functions, promoting tumorigenesis, metastasis, and chemoresistance [31]. ΔNp63α and ΔNp73α are isoforms expressed upstream of exon 3’, with distinct roles in cellular regulation [35]. While ΔNp63α orchestrates an epithelial cell-specific chromatin landscape and binds to numerous genome sites, ΔNp73α exists as an open tetramer competent to bind to DNA [36]. On the other hand, TAp63α and TAp73α, highly expressed in mammalian oocytes, play crucial roles in chromosome pairing and separation during meiosis [17]. Despite similarities in their transactivation-inhibitory (TI) domains, p73 isoforms remain constitutively tetrameric, while domain swap experiments demonstrate the capability of the p73 TI domain to support a closed state [5].

## 2. Overview of the Nuclear Pore Complex (NPC)

The nuclear pore complex (NPC), which consists of around 34 basic subunits, namely, nucleoporins (NUPs), serves as a sole remarkable gateway regulating molecular transport between the nucleus and cytoplasm [37,38,39]. Comprising non-polymeric protein complexes embedded within the double lipid bilayer membrane of the nuclear envelope, the NPC orchestrates the bidirectional, yet selective, passage of biomolecules essential for cellular function. Like a molecular sieve, the NPC imposes cargo discrimination based on size, shape, and specific recognition signals that make the process across the nuclear envelope precise and efficient. This machinery is very complex and serves not only for gene expression processes, signaling cascades, and DNA damage but also controls cellular responses to different physiological and pathological cues [40,41,42,43]. Given the multi-functionality of the NPC, extending beyond nucleocytoplasmic trafficking, it comes as no surprise that the NPC may be involved in the various mechanisms of diseases including cancer. This has been well documented in the pathogenesis of glioblastoma [44], ependymoma [45], squamous cell carcinoma [46,47,48,49], breast cancer [50], colorectal cancer [51,52], and prostate cancer [53,54], among others. Insight into the structure and functionality of the NPC is thereby demanded in unraveling fundamental aspects of cell biology while breaking new ground in the development of therapeutic strategies associated with dysregulation of nuclear pores in cancer.

**Figure 1:**
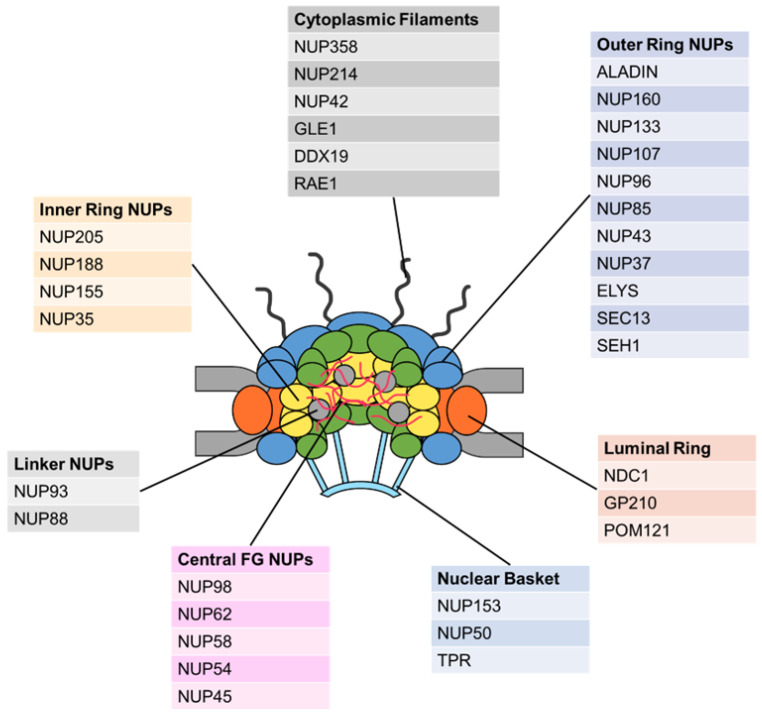
The schematic structure of the NPC. The structure of the NPC comprises the structural scaffold (inner ring, outer ring, luminal ring, and linker NUPs), the central channel (central FG NUPs), the cytoplasmic filaments, and the nuclear basket.

The assembly of the NPC represents a unique mechanism, wherein individual NUPs form distinct regions of the NPC, each with its own representative functions. The NUPs organize the NPC subcomplex into four groups: the structural scaffold, the central channel, the cytoplasmic filaments, and the nuclear basket (Figure 1) [55]. Indeed, each of the NUPs can further dynamically partition into both mobile and immobile sections. The introduction of immobile or static NUPs brings stabilizing elements that form a scaffold that can be converged with the nuclear ring (NR) and cytoplasmic ring (CR) to fit a more complex definition of the outer ring, alongside the well-defined inner ring (IR) and luminal ring (LR) on the plane of the nuclear membrane [56,57,58,59]. The NPC scaffold shows eightfold rotational symmetry. Each ring is considered to house between 8 and 16 individual subcomplexes. The NUP107-160 complex, known as the Y-complex due to its Y-shaped structure, constitutes the outer ring of the NPC on its nuclear as well as on its cytoplasmic faces, where it tags the nuclear basket and the cytoplasmic filaments, respectively [43].

As the core scaffold, the IR attaches itself to the CR, NR, and LR and is positioned at the convergence of the inner and outer nuclear membranes (INM and ONM, respectively) to directly form the central channel of the NPC. Harbored within the central channel of the NPC are a multitude of intrinsically disordered proteins (IDPs) termed FG-nucleoporins (FG-NUPs), which have unfolded structures engineered for dynamic interaction with transport receptors, creating a selective barrier critical for cargo transport [60,61,62]. A previous study reported that more than half of the FG-NUPs can form liquid-like condensates, a process in which FG motifs play an essential role. These motifs facilitate the formation of highly dynamic hydrophobic FG–FG cross-links, with interaction lifetimes shorter than 1 nanosecond, underscoring the rapid and transient nature of interactions within these condensates [63,64]. NUP98 in human is an essential FG-NUP which is associated with a certain type of leukemia where the FG-repeat domain of NUP98 fuses to a chromatin-binding domain, due to their capacity to concentrate biomolecules, especially oncogenic ones, into condensates [65]. Another study showed that more than 30% of the proteome was formed from IDPs, highlighting the potential to resolve disorder–function relationships of IDPs, which are frequently involved in various biological activities including cellular signaling, phase separation, aging, and viral entry [66].

The mobile NUPs, which play pivotal roles in physiological functions, comprise the cytoplasmic filaments, nuclear baskets, and permeability barriers formed by FG-rich NUPs. Typically, the mobile NUPs contain intrinsically disordered regions (IDRs) that form flexible regions to facilitate dynamic interactions with transport factors, cargo molecules, and other NUPs. These flexible regions support a rapid exchange, allowing transport receptors such as importins and/or exportins to bind to cargo molecules and translocate them across the nuclear envelope [67]. At the nuclear periphery, the basket helps target and preprocess nuclear export complexes, including mRNA protein complexes [37]. A recent study revealed that in the nuclear basket, Tpr and NUP153 are critical for mRNA export, while NUP50 has minimal impact. Specifically, Tpr is essential for docking mRNPs at the nuclear basket, and NUP153 regulates their transit through the NPC. Silencing of Tpr or NUP153 markedly affected the 3D export routes and reduced the efficiency of mRNA export. The study also showed a non-linear relationship between NUP153 copy number and mRNA export efficiency. Their findings provide insights into the distinct structural and functional roles of nuclear basket proteins in mRNA export [68]. Furthermore, RanBP2, also known as NUP358, is a part of the cytoplasmic filaments of NPC and an SUMO E3 ligase enzyme [69]. The two activities of RanBP2 in nucleocytoplasmic transport and SUMOylation were concentrated on in previous research [70,71]. Downregulation of RanBP2 was shown to be related to perturbing tumor progression in mice, which indicates that disruption in the SUMO pathway prevents malignancy [72].This has been deemed to be a consequence of the potential for direct and indirect regulation by SUMOylation of p53. Defects in the pathway might alter p53 activity, which is extremely important for tumor prevention and genomic stability [73].

The nuclear transport system is not limited to NUPs that form the NPC, but also comprises nuclear localization signals (NLSs), nuclear export signals (NESs), importins and exportins [74], Ran GTPase [75], post-translational modifications, protein conformation, and interactions with other proteins. The p53 protein shuttles through NPCs continuously to ensure appropriate cell responses. This shuttling activity is mediated by the NLSs and NESs within p53 itself, thus allowing it to respond to cellular signals as quickly as possible [76]. In the nucleus, p53 activates transcription to target genes involved in cell cycle arrest and apoptosis. Nuclear localization of p53 is required for gene transcription, whereas cytoplasmic p53 triggers apoptosis by directly activating pro-apoptotic molecules at the mitochondria [77]. This nucleocytoplasmic shuttling is essential for the tightly controlled regulation of p53 activities in modulating its activities between gene expression and apoptosis. This review is focused on p53 regulation through the NPC, since data regarding the regulation through nuclear transport elements other than the NPC has been reported elsewhere [78].

There have been continuous efforts toward the understanding of the structural and biochemical function of the NPC. Recent works describe how the evolutionarily conserved composite structures of the linker scaffold hold the outer ring, inner ring, and linker, and are critical to the maintenance of the integrity of the pore, yet do not constrain the plasticity of the central channel. Collectively, the data provide a blueprint for scrutinizing the complexities of NPC organization, both mechanical assembly and disassembly processes, the flexibility and fluctuation of NPCs, impact on transport, and anchoring mechanisms for asymmetric NUPs of both soluble and integral membrane cargoes. This comprehensive understanding promises to shed light on the origins of NPC-associated diseases, paving the way for elucidating their underlying causes [79].

## 3. p53 Family Regulation by NPC

### 3.1. NPC-Mediated Regulation of p53

Given the unique structure, composition, and flexibility of mobile NPC subunits, it is expected that they play broader functional roles in multiple regulatory processes independent of transport. Current studies have increasingly linked these NUPs to collaboration with p53 signaling pathways, marking their potential as therapeutic targets in p53-related pathologies. NUP210, for example, is mobile, dynamic, and highly expressed in many organs, including the brain, skin, lung, kidney, pancreas, and gut. Recent studies have highlighted the dynamic nature of NUP210, showing that its depletion does not affect nucleocytoplasmic transport but leads to the downregulation of several genes associated with differentiation via its luminal domain, which is continuous with the endoplasmic reticulum [61,80]. Additionally, the role of FG-NUPs and their interactions with receptors in the translocation of various protein molecules raises important questions regarding their potential involvement in the regulation of the p53 family.

The inactivation of p53 associated with loss of genomic integrity and multiple mechanisms contributing to genomic instability has frequently been reported in pancreatic ductal adenocarcinoma (PDAC) cases [81,82,83,84]. Aberrant expression of nuclear lamins and/or nuclear pores is a common event in the evolution of cancer, including PDAC [81,85]. However, the high likelihood of these hallmarks overlapping points out the need for comprehensive studies to finally define a druggable approach model that is tailored to the specific context underlying more complex pathological mechanisms. Recent research has found high-impact evidence in the interplay between the p53 regulatory network and nuclear architecture in advanced stages of PDAC [81]. Importantly, the study revealed the physical interaction between the components of the nuclear envelope NUP210 and lamin B1, representative of the NPC and the inner nuclear membrane, respectively, with the genomic loci of p53 to modulate gene expression. By generating chromosome enrichment analysis on p53-dependent genes associated with both the NPC and the nuclear lamina, researchers evaluated the shared gene sets to elucidate potential complementary regulatory mechanisms to uncover how p53, in conjunction with NPC components and lamin, orchestrates chromatin conformation and gene expression. Their approach suggested an integrated role in the regulation of p53-dependent genomic architecture and transcriptional activity. Interestingly, some of the genes impaired by the interaction of NPC, lamin B1, and p53 are also the genes commonly affected by copy number amplifications during cancer progression.These amplified genes, including the famous c-Myc, play critical roles in regulating the cell cycle, maintaining pluripotency, and coordinating cellular responses to many environmental cues in PDAC [81]. In another study, p53 was demonstrated to act as a suppressor of malignant transformation from premalignant pancreatic intraepithelial neoplasias (PanINs) to PDAC. PDAC can develop when pancreatic acinar cells undergo acinar-to-ductal metaplasia (ADM), leading to the PanINs that eventually progress to PDAC. Mild activation of p53 has the benefit of limiting the metaplastic transformation of acinar cells and dampening KRAS signaling (a driver oncogene) in PanINs, suggesting its potential as a treatment strategy [82,86].

Interactions of NUPs with the p53 pathway are apparent in the development of liver cancer in studies of hepatocellular carcinomas. Therefore, attention has been paid in the present study to the role of NUP155 with the p53 signaling pathway in relation to gene expression that might be significant in hepatocarcinogenesis. While the NUP155 is overexpressed in the HCC [87], the expression of p53 is typically present but functionally inactive due to factors like the hepatitis B virus (HBV) protein HBx inhibiting its wild-type function [88]. This functional inactivation of p53 can lead to p53 being genetically wild-type but functionally compromised in HBV-related HCCs [88,89]. p53 is also known to have a direct control on the repressed transcription of *NUP155* through its regulatory mechanisms [90]. This study established that p53-mediated repression resulted in low expression of NUP155 in HCC with wild-type compared to mutated p53, hence suggesting a role of p53 in controlling the transcription of *NUP155* in liver cancer [90]. More specifically, NUP155, in cooperation with the deacetylase enzyme HDAC4 [91], mediates translational repression of p21, one of the most important effectors of the p53 response, by manipulating the activity of FTSJ1, which is a master gene in the methylation of tRNA in human [90,92,93]. Both *NUP155* and *FTSJ1* are repressed by p53 and their expression levels are linked to the status of p53 in liver cancer [90]. This study revealed a network connecting translational control and the p53 pathway through NUP155. Moreover, the targeting of p21 in liver cancer was also demonstrated to be associated with the poor survival of patients through a feedback loop with NUP155. It was suggested that in hepatocellular carcinoma, there is a complex interaction of the p53–p21 axis with nuclear transport proteins [90]. Overall, the benefit of therapeutic intervention directed towards p53 will be taken into consideration only if there are specific mutations or dysregulations in the p53 pathway leading to a hypothesized restored tumor-suppressive function of the mutated p53 that increases the overall responsiveness to treatment. Additionally, NUP155 was identified as a key player in liver cancer progression under regulation by p53, making it another promising candidate for targeted therapy. Targeting NUP155 could disrupt the feedback loop between NUP155 and p53, restore the balance in the p53 network, and potentially enhance the tumor-suppressive functions of p53 to slow down the progression of HCC.

Interestingly, the regulation of p53 by the NPC is not confined to the capacity of mobile NUPs. Recent studies in glioblastoma brain cancer (GBM) have shown that NUP107, an NUP subunit that is part of the Y-complex of the rigid scaffold, interacts with the nuclear pore basket component NUP153, and plays a crucial role in p53 activation and stabilization [44]. NUP107 is identified as a potent oncogene in GBM, where it has been shown to be overexpressed alongside MDM2, a ubiquitin ligase known for its role in the proteasomal degradation of p53. The following result pointed out that NUP107 overexpression facilitates degradation of p53, mediated through the export factor dependent on the nuclear export signal (NES) to the NPCs. The NPCs, in turn, are associated with the 26S proteasome, facilitating the degradation of p53 within the nucleus [44,94,95] and promoting tumorigenesis by disabling the p53 function as tumor suppressor. Experimentally, depleted NUP107 disrupts this process, resulting in the stabilization and accumulation of p53 in the nucleus, which triggers a series of tumor suppressor responses detrimental to cancer cells. The ability of single NUP107 to stabilize p53 mostly relies on its capacity to provide the structural integrity of NPC [96,97]. The disruption of NUP107 promotes the disorder of other NUPs, including NUP153 and cytoplasmic filament NUP RANBP2. Positioned at the nuclear basket of the NPC, NUP153 tethers the 26S proteasome to the NPC [44,98], facilitating the efficient degradation of p53 at the nuclear periphery following p53 docking, assisted by the export factor XPO1. The loss of NUP153, arising due to the global disintegration of the whole NPC structure, is very similar in effect to the loss of NUP107, indicating the importance of maintaining NPC functional integrity with respect to the p53 degradation pathway. Modulation of p53 by NUP107 and NUP153 thus adds a nuanced layer of non-canonical regulation of tumor suppressors in cancer cells [44]. The ability of NUP107 and NUP153 as transport surveillance to influence p53’s stability and activity by dictating its nuclear export direction and subsequent degradation presents potential targets for therapeutic intervention. In glioblastoma, where alterations in the p53 pathway have been documented [99,100,101,102,103], impairing ongoing tethering of the p53 degradation machinery with the NPC may become an attractive novel approach for reactivating tumor-suppressive function in the p53 protein. This granular understanding of how the NUP107-NUP153-26S proteasome is involved in the regulation of p53 not only advances the understanding of nuclear transport dynamics but also suggests targets for the development of drugs that may perturb such a web of interactions, thus stabilizing p53 and allowing it to carry out its tumor-suppressive activities.

### 3.2. NPC-Mediated Regulation of p63 and p73

New studies have revealed that the regulatory role of NPCs is not only confined to p53 but also extends to other members of the p53 family, particularly p63 [46,48]. Through this perspective, NUP153 could be viewed as a strong ringmaster in gene expression regulation that exerts a critical impact on the p63 (*TP63*) transcription factor associated with squamous cell carcinoma tumorigenesis. Most recently, a study by Hazawa, et al [46] defined the rather intricate interaction of NUP153 and the transcriptional machinery regulating p63 expression. Subnuclearly, NUP153 resides at the nuclear periphery and serves as a major point of connection in chromatin organization and gene regulation. The regulation of p63 by NUP153 is mediated via the orchestration of super-enhancer (SE) regions. NUP153 strongly promotes the anchoring of SEs to the nuclear periphery, a mechanism critical for the efficient *TP63* mRNA processing from transcription to export. The anchoring mechanism is facilitated by the intrinsically disordered regions (IDRs) of NUP153, which engage in dynamic interactions with BRD4, a coactivator present at p63-associated SEs. The IDRs of NUP153 not only aid in localizing BRD4 to the periphery, but also stabilize the formation of phase-separated condensates, which are crucial for the high-level transcription characteristic of SEs. By mediating the positioning of p63 SEs at the nuclear periphery, NUP153 enhances the mRNA export rates, thereby boosting p63 expression levels. This is maintained in an active manner in the SCC cell oncogenic phenotype, in which p63 is able to transcriptionally activate genes that are important for cell proliferation and survival. NUP153 depletion led to a dramatic decrease in p63 expression that concomitantly resulted in impaired cellular proliferation and the induction of differentiation in SCC cells. This effect demonstrates a very important role for NUP153 in cancer cell biology when p63 expression is regulated, which goes on further to affect the whole of progression or response to therapy. The other aspect of NUP153 action on the regulation of gene expression pertains to general cellular architecture. It interacts with several members of the nuclear architecture, other NUPs, and chromatin remodelers for the precise tuning of spatial organization of important genomic loci. It likewise pertains to the process of gene expression at not only the transcriptional level but also at post-transcriptional levels through its effects on processes such as mRNA processing and export. To summarize, NUP153 acts to set the backbone for proper regulation of p63 in modulating SE dynamics and gene expression at the nuclear periphery [46]. These findings should be taken into account for possible therapeutic interventions such as the manipulation of the cascade of events concerning gene expression in cancer.

It has been appreciated that a single type of cancer can occupy multiple NUP expression profiles, which can serve as its particular biomarker. Especially, in another study in SCC, NUP62 overexpression is linked with the maintenance of an undifferentiated cellular state [48,104]. Overexpression of NUP62 in SCC might indicate a dependency on specific nucleocytoplasmic transport pathways that differ from overexpression of NUP153. Consequently, therapeutic strategies targeting these NUPs must be tailored to the specific molecular pathways of each condition. The complex interplay between NUP62 and the specific p63 protein isoform ΔNp63α was reported in that study. NUP62 is ubiquitously expressed in stratified squamous epithelia and shows further elevation in SCCs. The suppression of NUP62 results in the enhancement of differentiation markers, indicating its role in maintaining the proliferative and undifferentiated phenotype characteristic of malignant cells. The interaction between NUP62 and the ΔNp63α isoform, which acts as a key transcriptional regulator, is central to this mechanism. Importantly, the phosphorylation status of NUP62 is a key regulatory mechanism. The Rho-associated coiled-coil containing protein kinase (ROCK) pathway, known for its role in cell structure and movement, affects NUP62 by phosphorylating its FG-repeat regions. This post-translational modification (PTM) diminishes the interaction between NUP62 and ΔNp63α, leading to a reduction in the nuclear import of ΔNp63α. This reduction at the nuclear level highlights the dependency of p63 on NUP62 for effective nuclear localization and function. The study also effectively links ROCK pathway activity with the nuclear transport mechanisms of p63, providing a potential therapeutic target for modifying the behavior of SCC cells through the NUP62–ΔNp63α interaction or the regulatory actions of the ROCK pathway [48].

The regulation of p73 by the NPC is an aspect of cellular biology that has received relatively little attention in the scientific literature [105,106]. Despite the extensive studies of p73 and NPC individually, their interaction remains largely unexplored. However, a recent study utilized HCC-induced zebrafish models successfully to cover the role of nucleoporin ELYS, encoded by the *AHCTF1* gene, and its association with the p53 family of proteins, including p53, p63, and p73, albeit indirectly via the induction of the DNA damage response [105]. The research concluded that a 50% decrease in the dosage of the *AHCTF1* gene mediated a permissible, significant reduction in liver volume only in a cancer-associated state but had no effect on the non-hyperplastic tissues. This reduction in the activity of the *AHCTF1* gene also reduced the formation of nuclear pores, the mitotic spindle assembly, and chromosome segregation, which ultimately led to DNA damage and activation of the *TP53*-dependent transcriptional program, leading to induction of cell death and cell cycle arrest. The study also demonstrated that *AHCTF1* heterozygosity, in combination with reduced expression of another NUP gene, *RANBP2*, or treatment with the nucleocytoplasmic transport inhibitor Selinexor, effectively blocked *KRAS*-driven hepatocyte hyperplasia in zebrafish models of HCC. Gene expression analysis of patient samples from the liver hepatocellular carcinoma dataset in The Cancer Genome Atlas revealed a positive correlation between high expression of NUP107–160 complex components, including *AHCTF1*, and worse overall survival in HCC patients. Furthermore, the paper highlighted the potential of targeting NPCs as a promising therapeutic approach for HCC. Analysis of mRNA expression levels of NUP107–160 complex components in HCC patient samples showed that overexpression of these genes was associated with poorer overall survival, while under-expression correlated with better survival outcome. Mild depletion of ELYS was shown to disrupt cellular processes, leading to the accumulation of DNA damage and activation of *TP53* transcriptional programs, ultimately inducing cell death and cell cycle arrest in hyperproliferative oncogene-expressing cancer cells [105]. Collectively, these insights into molecular mechanisms suggest that combined treatments targeting NUPs could offer effective strategies to trigger p53-, p63-, and p73-mediated cellular responses to oncogenic stress, thereby combating HCC and improving patient outcomes.

### 3.3. Regulation of p53 Target Genes by the NPC and Its Implications for the p53 Network

Of importance, the alteration of p53 target genes may strongly influence the transcription factor p53 itself, thus influencing the stability, subcellular localization, and activity requisite for this protein to effectively function as a tumor suppressor. In this manner, NUPs could exert a feedback response over p53 regulatory networks via altered expression of genes controlling the cell cycle or apoptosis, such as the p21 (*CDKN1A*) gene. Specifically, the upregulation of p21 can stabilize p53 by inhibiting its degradation pathways [2,107,108]. Overexpression of certain NUPs which contribute to the development of cancer has been increasingly recognized in the field of oncology. However, depending on the context, the function of the NPC or its specific subunits can also be considered as suppressing malignancy, particularly when their mutations or silencing occur in the origin of certain cancers [109,110,111]. This bifunctionality of NUPs illustrates their very complex role in cancer biology since they act as oncogenes or tumor suppressors depending on the level of their expression and interaction in the intra- or extracellular environment. Earlier findings indicated that NUP98 may also have a role as a tumor suppressor. Beyond its basic role in transport, NUP98 has also been implicated not only in mRNA export but also in its interaction with transcriptionally active chromatin, highlighting its importance in gene regulation [112]. In the context of p53 target gene regulation, NUP98 has a unique role in selectively regulating the expression of p21 in human hepatocellular carcinomas (HCCs). This regulation is not at the level of transcription initiation but rather at a post-transcriptional stage. For example, NUP98 enhances the levels of p21 by stabilizing its mRNA: preventing it from being degraded by the exosome complex. In this manner, NUP98 stabilizes mRNA by interacting with the 3’ untranslated region of a messenger RNA and protecting it from rapid degradation in the cell. Concomitantly, a significant decrease in the levels of p21 (*CDKN1A*) mRNA and protein has been observed with the depletion of NUP98, especially upon activation by p53. Taken together, all this can be translated into the fact that NUP98 is indeed one of the key players in mediating all these effects of p53 in response to stress, which includes DNA damage. This is significant as p21 is one of the critical effectors of the p53 pathway, mediating cell cycle arrest and apoptosis, and therefore, is central to the tumor suppressor functions of p53. Moreover, lower levels of NUP98 in these cancers also correlate with a diminished expression of p21, pointing to an important role for NUP98 in maintaining proper function of tumor suppressor pathways mediated by p53. Overall, these data suggest that NUP98 is likely to act as an adaptive regulator of p53 target genes through action at a mechanism separated from its role in nucleocytoplasmic transport. Its ability to stabilize specific mRNAs like p21 (*CDKN1A*) highlights a sophisticated layer of gene regulation that integrates the NPC with the core components of cell cycle control and apoptosis, offering insights into novel cancer therapeutic strategies to prevent HCC progression [109].

**Figure 2:**
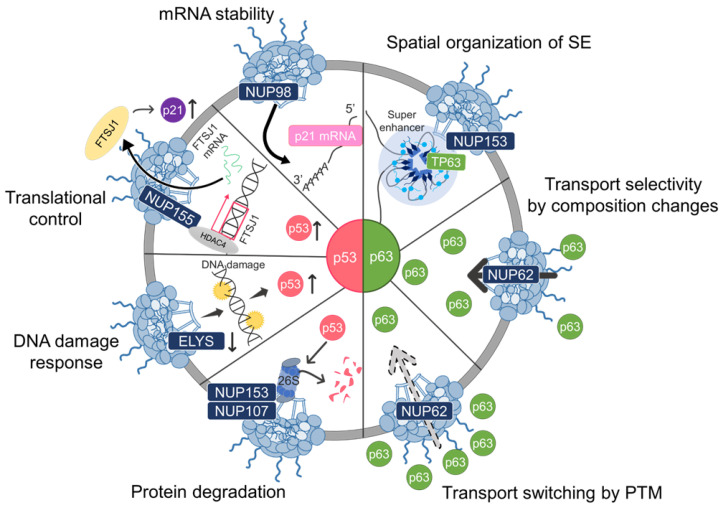
The role of NPC in p53 family regulation. Several NUPs are involved in the regulation of p53 and/or p63 each with its distinct mechanism. NUP107, together with NUP153, acts as a p53 surveillance mechanism by ensuring the proteasome degradation system in the nuclear periphery [44]. NUP153 anchors SEs to the NPC to enhance *TP63* expression [46]. NUP62 has two roles in maintaining p63 regulation: transport selectivity through compositional changes and transport switching via PTM [48]. NUP155 regulates the mRNA translation of p21 (*CDKN1A*) as part of the p53 network [90]. DNA damage induced by ELYS depletion triggers the activation of p53 transcription programs [105]. NUP98 regulates p21 (*CDKN1A*) mRNA levels through a PTM and forms a complex that protects the mRNA from degradation by the exosome (mRNA stabilization) [109]. Arrows ↑: upregulated, ↓: downregulated.

To date, contemporary studies have revealed an unforeseen adaptability of NPCs in the activation and stabilization of the p53 family. This NPCs’ spatio-temporal regulation engages through various specific mechanisms including, but not limited to, post-translational modification (PTM), DNA damage response, mRNA stability, and protein degradation (Figure 2), highlighting the significance of NPC and its regulators as critical parts of targeted cancer therapies.

### 3.4. Structural Insights into p53 Family Regulation by NPC

The study of the NPC, while extending beyond its role as a sole molecular gateway between the nucleus and cytoplasm, reveals an enormously complex structure. This complexity corresponds to the massive protein assembly composed of multiple NUPs [113,114], which are divided into parts: those responsible for maintaining the structural integrity of the NPC itself and those directly or indirectly involved in gene regulation. It is known that proteins belonging to the p53 family constantly shuttle through the NPC [115], and several non-canonical regulatory mechanisms controlled by the NPC have begun to emerge, as we previously discussed. Several studies indicate that the regulation of the p53 family can be selective, prioritizing certain NUPs over others. An insight into the NPC structure in relation to p53 family regulation would remain incomplete without addressing the critical aspects of p53 compartmentalization and proper localization [74,116]. More interestingly, some components of the NPC structure are oriented towards the cytosol, and others toward the nucleoplasm. This depiction consequently allows p53 to interact with different sets of proteins in the cytoplasm and nucleus [96,117]. Following stress signals by DNA damage, for example, p53 is stabilized and starts accumulating in the nucleus, where it activates genes involved in these stress responses [118].

Therefore, how crucial are the structural elements of the NPC in shaping the p53 family’s cellular fate? Each component of NPC has unique functions that contribute to the regulation and stabilization of p53 proteins. The central core of the NPC facilitates the selective transport of p53 between the nucleus and cytoplasm, ensuring proper localization and function. However, NUP98, which is located in this central channel, performs an additional role in mRNA stabilization of p53 target genes [109]. RanBP2, as part of cytoplasmic filaments, promotes the p53 regulation by SUMOylation [10]. Post-translational modifications (PTMs) of NUPs within the NPC can impact p53 stability and function, highlighting the NPC’s role beyond mere transport [47,109]. The physical distance of NUP153 from other cellular components, such as SEs, is a determinant factor in p63 regulation, enhancing the expression of p63 in a spatio-temporal manner [46]. The NPC’s structural integrity is vital for the precise regulation of the p53 family. Not only can NPC disintegration perturb the normal transport of p53, but interestingly, current studies have discovered that the proteasome, as an important degradation system tethered to the NPC, efficiently inactivates the p53 intranuclear protein [44]. Detailed structural insights into the NPC can pave the way for targeted cancer therapies, emphasizing the importance of maintaining NPC functionality in cellular homeostasis and tumor suppression.

## 4. Therapeutic Implications—Challenges and Future Directions

### 4.1. Overview of Classical Therapy

Generally, therapies to target abnormalities in p53 depend on whether the p53 abnormality belongs to the mutational or non-mutational factor [119,120,121]. Given that wild-type p53 inhibits a wide range of tumors while mutant p53 promotes cancer development, current studies focus on developing therapeutic strategies to restore p53 function [122].

In cases where p53 is non-mutated but inactivated due to excessive regulation by MDM2, therapeutic strategies aim to disturb the p53-MDM2 interaction: several studies have come into focus recently, such as the study on nutlin-3a, which is a potent and effective enantiomer of the racemic nutlin-3. Nutlin-3a links to the hydrophobic pocket of MDM2 in competition with p53, thus inhibiting MDM2-mediated p53 ubiquitination and degradation [123,124,125,126]. Consequently, this stabilizes and accumulates p53 in the cell, thereby activating a p53-dependent program of transcription that includes the upregulation of genes for cell cycle arrest and initiation of DNA repair and apoptosis. This activation subsequently arrests cell division through tumor suppressor pathways and triggers programmed cell death in cancer cells with wild-type p53, ultimately decelerating tumorigenesis [127,128,129].

Nutlin-3a is one of the compounds that within preclinical studies showed great promise with respect to anticancer activity and is currently under investigation as a potential therapeutic agent in the treatment of various human malignancies, most of which are found with wild-type p53 function [130,131]. However, it has to be noted that Nutlin-3a may not be effective in tumors with p53 mutations or alternations preventing p53 functioning, hence suggesting the need for personalized therapeutic modalities depending on p53 status [119,124]. Another major problem reported by recently emerging studies is that Nutlin-3a often leads to cell cycle arrest, which is reversible, and thereby, reduces its therapeutic utility. In such instances, Nutlin-3a may even shield cancer cells from standard chemotherapy, which depends on cell cycle progression. Additionally, MDM2 inhibitors may display on-target clinical toxicity and can predispose cells to genomic instability. Therefore, for leveraging p53 activation as a cancer therapy, it is crucial to identify compound combinations that reduce the dosage of MDM2 inhibitors needed, while also enhancing their capacity to induce cell death rather than inducing reversible cell cycle arrest [127].

*TP53* mutations are highly prevalent in a wide variety of human cancers and have often been found to lead to a double-edged effect: loss of crucial tumor-suppressive functions and gain of traits that fuel cancer growth, for which reactivation of the mutant p53 is required for recovery to normal functionality of the p53 protein. Since these mutations predominantly manifest within the DBD of p53, resulting in the attenuation of its tumor suppressor capabilities including DNA binding and the transcriptional activation of target genes, reactivation strategies seek to rectify the misfolded structure of mutant p53. Lately, studies have expanded upon previous approaches to rescue mutant p53, focusing on reactivation through structural manipulations and peptides. Notably, a substantial number of mutants, encompassing both DNA contact and structural hot-spot variants such as R273H, R273C, R248Q, R282W, and G245S, have exhibited potential for DNA binding activation through C-terminal manipulations [119,132,133,134,135,136].

Recently, a study by Kevan M. Shokat’s team successfully established a therapy that directly targets mutant p53 to rescue wild-type function. The research pinpointed the covalent compound KG13, capable of specifically interacting with the p53 somatic mutant cysteine Y220C. The p53 somatic mutant cysteine Y220C is a specific mutation in the p53 protein, where the tyrosine (Y) at position 220 is replaced by cysteine (C). This mutation occurs within the DBD of p53 and results in a loss of thermal stability, which indirectly inhibits the protein’s ability to bind DNA and regulate target gene expression. This destabilization leads to the loss of p53’s tumor-suppressing functions, contributing to cancer progression. KG13 works by covalently binding to the mutant cysteine at position 220 (Y220C) of the p53 protein. This binding stabilizes the mutant protein, restoring its thermal stability to levels comparable to the wild-type p53. As a result, KG13 reactivates the tumor-suppressing functions of p53 by enabling it to properly regulate target genes involved in cell cycle control and apoptosis [137,138].

The main potential of targeting TAp63 and TAp73 is to induce apoptosis and cell cycle arrest by activation of the p53-responsive genes involved in these processes, such as p21 (*CDKN1A*)/*Waf1*, *Bax*, and PUMA (*BBC3*) [139,140]. For example, they can be selectively targeted in tumor cells to perform the apoptosis at a large scale by disturbing its interaction with the inhibitors of the apoptosis-stimulating protein of p53 (iASPP) [141,142]. Similarly, TAp63 can activate the promoters of the genes involved in both apoptosis and cell cycle regulation. In contrast, drugs targeting ΔNp63α and ΔNp73α are focused on their dominant-negative properties. These isoforms inhibit the transcriptional activities of TAp63, TAp73, and p53, contributing to chemoresistance and anti-apoptotic effects. For example, ΔNp73α and mutant p53 can upregulate ΔNp63, which further leads to the induction of chemoresistance. Apart from this, ΔNp73α can also inhibit TAp73 and TAp63 using mechanisms involving promoter competition and heterocomplex formation [143]. Generally, whereas TAp63 and TAp73 are targeted for promoting apoptosis and tumor suppression, ΔNp63α and ΔNp73α are targeted to overcome their inhibitory effects and reduce chemoresistance. Another study showed that prodigiosin facilitates p73 upregulation by disrupting its interaction with mutant p53, inducing p73’s WTp53-like transcriptional activity and anti-tumor potential through p21 activation. Compounds such as RETRA and short interfering mutant p53 peptides (SIMPs) also restore p73 function by disrupting its interaction with mutant p53. In neuroblastoma, 1-carbaldehyde-3,4-dimethoxyxanthone (LEM2) prevents mutant p53’s inhibition of TAp73α, a tumor-suppressive C-terminal splice variant, by disrupting both mutant p53 and MDM2 binding to p73, thereby enhancing p73 function [31,144].

### 4.2. The Development of Combinatorial Therapy

Leveraging personalized medicine based on individual cancer profiles allows for a tailored treatment regimen that maximizes efficacy and reduces adverse effects. Employing a combinatorial therapy is a preferable strategy as it provides greater selectivity for targets relevant to the underlying mechanism of pathogenesis. The p53 alteration often derives from a more complex mechanism that includes interaction with multiple signaling pathways, from mRNA processing, to cellular trafficking, until degradation. Therapeutic with combinations of several targets or therapies that inhibit multiple pathways effectively address these multi-dimensional interactions. Such an approach may also counter the mechanisms of resistance that are generally observed with single-agent therapy by causing damage through a multiplicity of redundant or compensatory routes, and thus, improving the overall therapeutic benefit.

Direct targeting of the NPC to change cell function, and thus, cure disease, has been declared technically infeasible because the NPC is too fundamentally important to the cells. However, that perspective has begun t change as several studies have begun to demonstrate that cancer cells have a stronger reliance on NPCs than normal cells, highlighting a unique vulnerability that can be exploited for cancer treatment [145,146,147,148]. Cancer cells and normal cells have different responses to the inhibition of the NPC assembly. In contrast, inhibition of NPC formation markedly leads to rapid cell death in cancer cells, while normal cells experience a reversible cell cycle arrest. Recently, a study by Sakuma et al. [148] demonstrated a significant cell death in the A375 melanoma and HT-29 colorectal cancer cell upon inhibition of the NPC assembly. Consistently, the SubG1 population was markedly suppressed, along with an increase in several apoptotic markers, indicating the greater importance of NPCs for their survival. In contrast, normal cells such as IMR90 fibroblasts and RPE1 retina pigment epithelial cells undergo cell cycle arrest in the G1 phase. The arrest is reversible; once the formation of NPCs has been re-established, the cells start to proliferate normally, suggesting that normal cells are relatively less dependent on NPCs compared to their malignant counterparts. In general, the sensitivity and selectivity of these cancer cells make targeting the NPC aberration a promising therapeutic strategy to improve outcomes while minimizing toxic effects on normal cells [148].

NUP155 has been associated with the regulation of gene expression, especially HCC. In HCC, p53 is always expressed but functionally inactive due to reasons like hepatitis B virus protein HBx. It has been shown that p53 suppresses NUP155 transcription, which plays an important role in keeping NUP155 levels low in wild-type p53 HCC compared to mutated p53 HCC [90]. From a therapeutic standpoint, relation with NUP155 in HCC would prevent this feedback loop between NUP155 and p53 to restore p53 network balance and intensify its tumor-suppressive function. The second strategy is through targeting the regulation of p21 (*CDKN1A*) by NUP155, where such an interaction mediated by the deacetylase enzyme HDAC4 and methylation of rRNA by FTSJ1 could be modulated to confer improved p53 response against liver cancer progression.

In glioblastoma, NUP107 is highly expressed and exhibits a very close relationship with NUP153 in the degradation of p53 and tumorigenesis. A study demonstrated that p53 degradation by NUP107 overexpression requires NES-mediated export, which is assisted by XPO1 activity, and the 26S proteasome, which is anchored at the NPC by NUP153. An interference with this process stabilizes p53 and triggers tumor suppression responses that are lethal to the cancer cells [44]. Therapeutic approaches would also be directed toward the interactions of NUP107, NUP153, and the 26S proteasome in their effort to prevent p53 degradation and enhance its tumor-suppressive activities. This could encompass small-molecule inhibitors that can disrupt the interaction between these proteins or RNAi methods to silence NUP107 and NUP153 expression.

NUP153 is also involved in the mechanism that regulates p63 expression, making it highly relevant to squamous cell carcinoma. As such, NUP153 affects high-level transcription of p63-related genes by tethering SE regions to the periphery of the nucleus. Targeting NUP153 in SCC could produce an opposite effect: a reduction in p63 expression, cellular development, and differentiation [46]. A new possibility of a therapeutic approach of this disease is opened, since it is now indicated that NUP62 acts in an undifferentiated state by the interaction of ΔNp63α, meaning that its separation could promote differentiation but not tumorigenesis [48]. Therapeutic strategies could be aimed at the ROCK pathway, which mediates NUP62 phosphorylation and its association with ΔNp63α.

The abnormal levels of NUPs that are integrated in the regulation of the p53, p63, and p73 pathways show complex potential in regard to the possibility for therapeutic targeting of these interactions in cancer. Since the NUPs play cell-specific roles within several types of cancer, therapeutics strategies should target molecular pathways and relevant interactions for each condition. In HCC, targeting NUPs like ELYS and RANBP2, combined with nucleocytoplasmic transport inhibitors like Selinexor, has shown promise in preclinical models [105]. This would disturb the formation of nuclear pores and the sequestration of chromosomes, showing that DNA damage and activation of p53 are initiated to promote cell death of hyperproliferative cancer cells. For those cancer types in which more than one expression profile of NUPs is present, the understanding of which specific roles are being executed by NUPs will help to develop targeted therapies. The differential roles for NUP153 and NUP62 in regulating p63 offer an alternative therapeutic approach that provides more nuanced therapies against SCC.

### 4.3. Implications of Combinatorial Therapy and Identification of Treatment Response

Several implications for cancer treatment arise from combining drugs that directly restore p53 function with those targeting the NPC components responsible for p53’s distinctive mechanistic regulation. Such implications include enhanced drug efficacy and selectivity [149,150,151], overcoming drug resistance, synergistic effects, reduced toxicity, and broader applicability. Modification of NPC components can result in enhanced p53 nuclear cytoplasmic transport, and thus, assure its proper localization in the first place, therefore improving its tumor suppressor function. This will increase both efficacy and specificity for treatment. Moreover, there is a better chance of dual therapies overcoming drug resistance, which commonly develops in single-agent therapies due to the mutation of target proteins or activation of compensating survival pathways, as reported in several studies [152,153,154,155,156]. One of the key objectives for designing and testing combination drug strategies is to derive synergistic effects, whereby the impact of a combination is much more significant than that predicted by the sum of the impact of each drug [157,158,159,160,161]. The synergy can eventually lower toxicity by enabling the use of individual drugs at reduced dosages while maintaining therapeutic efficacy [149,162,163]. Finally, targeting NPC components in conjunction with p53 restoration has the potential to be broadly applicable across different cancer types. Given the fundamental role of NPC in cellular transport and the ubiquitous importance of p53 in tumor suppression, this strategy can be effective in various malignancies, including those with diverse genetic backgrounds and tumor microenvironments. This broad applicability makes it an attractive option for developing universal cancer therapies.

Advances in molecular techniques have primarily contributed to precision oncology by developing sensitive and specific biomarkers aided by novel analytical methods [164,165]. It has been defined that the detection of DNA, RNA, protein, or transcription factors resulting from the primary tumor or as the host response to the presence of a tumor gives predictive and prognostic information guiding patient care. It is further worth considering that for personalized medicine, highly detailed on-treatment biomarker monitoring would be necessary. Using molecular biomarkers in therapies targeting the NPC and p53 stabilization would be essential to monitor and assess early treatment response, treatment failure, or disease progression/relapse. Building on previous studies, some methods including immunohistochemistry (IHC), immunofluorescence (IF) [166,167], fluorescence in situ hybridization (FISH), polymerase chain reaction (PCR), next-generation sequencing (NGS) [168], and gene expression profiling (GEP) can also be applied to cancers with p53-NPC defects [164] and could provide valuable biomarkers. For example, IHC and IF can be employed to visualize the localization and expression levels of NPC components and p53 in tumor tissues. Therefore, the changes in the nuclear cytoplasmic distribution of p53 could be directly assessed. A recent study demonstrated the use of nanoparticles, specifically nanodiamonds, to deliver miR-34a to enhance the levels of p53 and its acetylated form in the MCF7 breast cancer cell line [169]. In addition to IF, a label-free tomographic technique offers promising outcomes for identifying treatment response in nano-therapy by allowing the visualization and quantification of nanoparticles, including nanodiamonds, within biological systems without fluorescent labeling, thereby preserving the native state of cells and tissues [170]. Moreover, circulating tumor DNA (ctDNA), a small DNA fragment derived from tumor cells, which contains tumor-related genomic information, is an ideal biomarker for real-time monitoring of tumor progression [171,172]. A previous study in metastatic castration-sensitive prostate cancer (mCSPC) patients showed that the combination of ctDNA and tissue revealed gene-level changes, including broad *TP53* mutation and *MSH2* gene-truncating mutation [173]. Here, they successfully evaluated tumor molecular subtypes, providing a solid foundation for the development of the next targeted treatment strategies for cancer patients [173,174]. In tumors with p53-NPC defects, the changes in ctDNA levels of p53 target genes and NPC components could, therefore, also indicate the tumor response to therapy.

## 5. Conclusions

In summary, the spatio-temporal organization of p53 family genes by the NPCs discussed in this review illuminates how cancer cells co-opt the structure of the nuclear periphery to regulate p53 family gene signaling. Even though this review focuses on a limited number of cancer types and a selected group of NPC subunits, the cumulative evidence underlines the involvement of various NUPs in a wider array of cancers. Therefore, in the near future, expanding studies of interplay between NPC components and p53 pathways across more cancer types will be instrumental for the development of the oncology and therapeutic fields of study.

## Data Availability

Not applicable.
